# Simplified drug efficacy screening system for sleep-disorder drugs using non-human primates

**DOI:** 10.1016/j.heliyon.2020.e03524

**Published:** 2020-03-04

**Authors:** Keita Sakai, Akiyoshi Ishikawa, Yuri Mizuno, Takehiro Maki, Yasuhiro Oda, Eiki Takahashi

**Affiliations:** a Sleep Science Laboratories, HAMRI Co., Ltd., Ibaraki, 306-0128, Japan; b Bio-X Institutes, Shanghai Jiao Tong University, Shanghai, 200240, People's Republic of China; c Research Resources Division, RIKEN Center for Brain Science, Saitama, 351-0198, Japan

**Keywords:** Neuroscience, Physiology, Zoology, Animal physiology, Veterinary medicine, Animal behaviour, Cynomolgus monkey, Nano-Tag, Screening, Sleep disorder drug, Telemetry

## Abstract

The most widely used animal models to develop sleep-disorder drugs are rodents, particularly rats and mice. However, unlike humans, these rodents are nocturnal. Thus, diurnal non-human primates represent a valuable and more translational animal model to study sleep. Although sleep-disorder drugs have been screened in non-human primates, the use of a telemetry system is not a desirable method for a rapid drug efficacy assessment system because of the need for expensive equipment, complicated surgery, and the long time before results can be obtained from analysis by inspection. Locomotor activity has traditionally been used as an indicator of the effects of drugs, genes, and disease models. The Nano-Tag, a new device for analyzing activity after an easy implantation surgery, measures locomotor activity without expensive equipment and the need for inspection for data processing, and the overall cost is much lower than that of a telemetry system. In this study, we compared the data obtained from polysomnography and on locomotor activity in telemetry transmitter-embedded cynomolgus monkeys by implanting the Nano-Tag subcutaneously in the forehead and administering sleep-disorder drugs to confirm if sleep–wake states could be measured using the Nano-Tag. When we compared the changes in awake time per unit time measured using polysomnography and locomotor activity counts per unit time measured using the Nano-Tag, cynomolgus monkeys exhibited a diurnal preference, and the correlation coefficients were positive during the 24-h period. Additionally, the correlation coefficients during the 12-h dark period were positive when the hypersomnia treatment drug modafinil was administered. The correlation coefficients during the 12-h light period were also positive when the insomnia treatment drug triazolam was administered. These results suggest that measuring locomotor activity is an effective tool for identifying sleep–wake states and screening sleep-disorder drugs at low cost and with less burden to animal subjects.

## 1 Introduction

Sleep disorder is a significant social problem in stressful societies. The causes and the mechanism of the pathogenesis of sleep disorder are so complicated that treatment approaches differ in each case, yet treatment drugs are indispensable in every case. Drugs, including modafinil, pemoline, or methylphenidate, are administered for hypersomnia treatment [1]. The insomnia treatment drugs are often called sleep inducers or simply sleeping drugs; these include drugs that affect GABAA receptors, such as barbital, triazolam, and zolpidem [2]; melatonin receptors, such as ramelteon [3]; and orexin receptors, such as suvorexant [4]. Muscle relaxant action, defects in memory, addiction, and resistance are reported side effects of sleep-disorder treatment drugs [5]. As the frequency of prescribing sleep-disorder treatment drugs has risen, the development of safer drugs applicable for the long-term is in immediate demand.

Experiments on model systems are essential for developing novel drugs and understanding sleep mechanisms. Various animals such as zebrafish [6], mice [7], rats [8], cats [9], dogs [10], and monkeys [11] are used to develop sleep-disorder treatment drugs. The most frequently used laboratory animals among them are mice and rats, rodents that are polyphasic sleeping animals—i.e., they are nocturnal and sleep several times per day [12, 13]. Hence, mice and rats are different from humans, which are diurnal animals with a monophasic sleeping pattern—i.e., they sleep once per day [14]. Monkeys also sleep several times a day, but they are also diurnal animals [15] and exhibit the most similar sleeping pattern to humans compared to other experimental animals. When conducting a sleeping experiment where activities of drug-metabolizing enzymes differ among animal species [16], and particularly when an analysis regarding addiction is concerned, sufficient consideration of the test animal model is mandatory. Therefore, monkeys, which are primates, would be a suitable animal model for experiments related to sleeping. The different types and stages of wake and sleep can be identified using polysomnography, which simultaneously measures several body functions using electroencephalography (EEG), electromyography (EMG), and electrooculography (EOG). Polysomnographic data have been recorded from non-human primates using telemetry implants [17, 18]. Although telemetry is a remote system that enables the electronic capture of biological signals in freely moving animals, which leads to reduced stress and changes in sleep characteristics [15], telemetry involves complex surgical implantation of electrodes that are relayed to a biosensor device (transmitter) positioned subcutaneously, intramuscularly, or intraperitoneally. Sleep architecture follows an alternating cycle of rapid eye movement (REM) and non-rapid eye movement (NREM) sleep throughout a typical night and is defined in the laboratory by recording the electrical field activity of large groups of neurons and muscle cells in humans and model animals [19, 20]. By contrast, an implantable locomotor activity measuring device, such as a Nano-Tag (ACOS. Co., Ltd., Nagano, Japan), is minimally invasive to animals because it is small (17.9 mm diameter and 2.5 g weight) and can be subcutaneously implanted during a relatively easy surgery [21]. It does not require expensive equipment, the data can be processed with the accompanied software, and the overall cost is much lower than that of telemetry.

In this study, to confirm if the sleep and wake states could be measured and if activity measurements could be used to develop a sleep-disorder drug-screening system, we obtained measurements from telemetry transmitters and Nano-Tags embedded in cynomolgus monkeys and compared the data obtained from polysomnography and on locomotor activity after administering representative hypersomnia and insomnia treatment drugs.

## 2 Materials and methods

### 2.1 Ethical declaration

This research was conducted in accordance with the Declaration of Helsinki and was approved by the Animal Experiments Committee of HAMRI Co., Ltd (Ibaraki, Japan, Approved number: 18-H074).

### 2.2 Animals

Cynomolgus monkeys (male, 3–4 years old, 3.0–5.0 kg body weight) from Apes Inc. (Ibaraki, Japan) were housed in individual stainless steel cages (CyM2016, W550 × H815 × D735 mm, Shintoyo Seisakusho, Ltd.) and allowed access to tap water and food pellets (PS-A, Oriental Yeast Co., Ltd., Tokyo, Japan). Cynomolgus monkey were maintained at 24 ± 3 °C and 55 ± 25% humidity under a 12-h light/dark cycle (light period: 7:00–19:00 h).

### 2.3 Surgery

**Figure 1:**
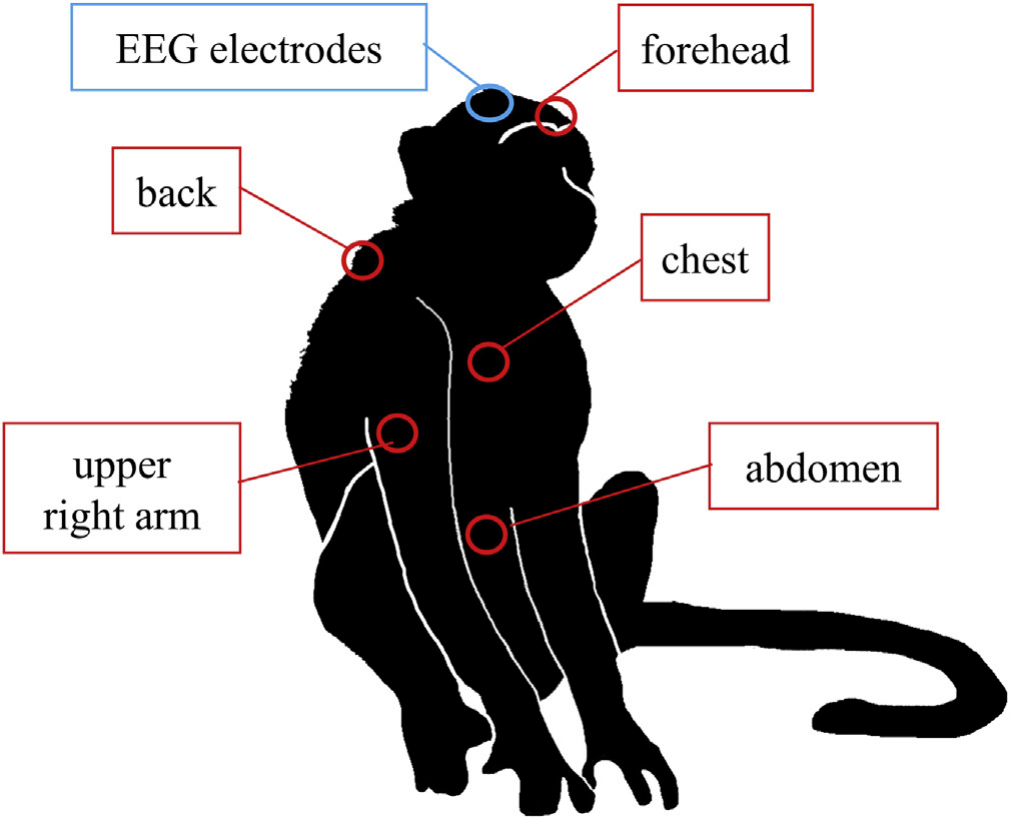
Implantation sites of EEG electrodes and Nano-Tags for measuring locomotor activity in cynomolgus monkeys. EEG, electroencephalogram.

A telemetry transmitter (TL10M3-D70-EEE; Data Sciences International, New Brighton, MN, USA), which consisted of a three-channel biopotential device for measuring EEG, EOG, and EMG signals, was implanted intraperitoneally into each subject according to our previous report [15]. A reference electrode (anterior/posterior to bregma, −15.0 mm; lateral to midline, −5.0 mm) and two EEG electrodes (anterior/posterior to bregma, −5.0 mm; lateral to midline, +5.0 mm; anterior/posterior to bregma, −15.0 mm; lateral to midline, +5.0 mm) were screwed into the skull and two EOG electrodes were affixed at the level of the orbital arch bone. Nano-Tags were implanted in the foreheads of two monkeys and in the forehead, back, chest, abdomen, and upper right arm of one monkey (Figure 1) at least 2 weeks after the telemetry transmitter implantation surgery. The monkeys were anesthetized using 5 mg/kg ketamine (Ketalar; Daiichi Sankyo Propharma Co., Ltd.) and 0.02 mg/kg medetomidine (Domitor; Nippon Zenyaku Kogyo Co., Ltd., Tokyo, Japan)**.** The skin was incised, a Nano-Tag was implanted subcutaneously at each location, and the skin was sutured with suture thread (Alfresa Pharma Co.).

After the surgery, the monkeys were administered analgesics (lepetan and buprenorphine hydrochloride; Otsuka Pharmaceutical Co., Ltd., Tokyo, Japan) and an anti-inflammatory agent (prednisolone; Kyoritsu Seiyaku Corp., Tokyo, Japan) and an antibiotic (penicillin G potassium; Meiji Seika Pharma Co., Ltd.).

### 2.4 Telemetry transmitter and Nano-Tag data collection

The monkeys were moved to individual monitoring cages (CyM2016; Shintoyo Seisakusho Ltd., Chiba, Japan). EEG, EOG, and EMG signals were collected via a receiver (RMC-1; Data Sciences International) and Dataquest ART software (Data Sciences International). The locomotor activity data stored by Nano-Tag were transferred to the Nano-Tag/Viewer program (Kissei Comtec Co., Ltd., Nagano, Japan) using a FeliCa reader (RC-S360; Sony Corp., Tokyo, Japan) within 1 cm of the implant. Behavior was recorded using an infrared camera (VHC-IR982W; Takenaka Engineering Co., Ltd., Kyoto, Japan).

### 2.5 Analysis of telemetry system data on sleep–wake states

**Figure 2:**
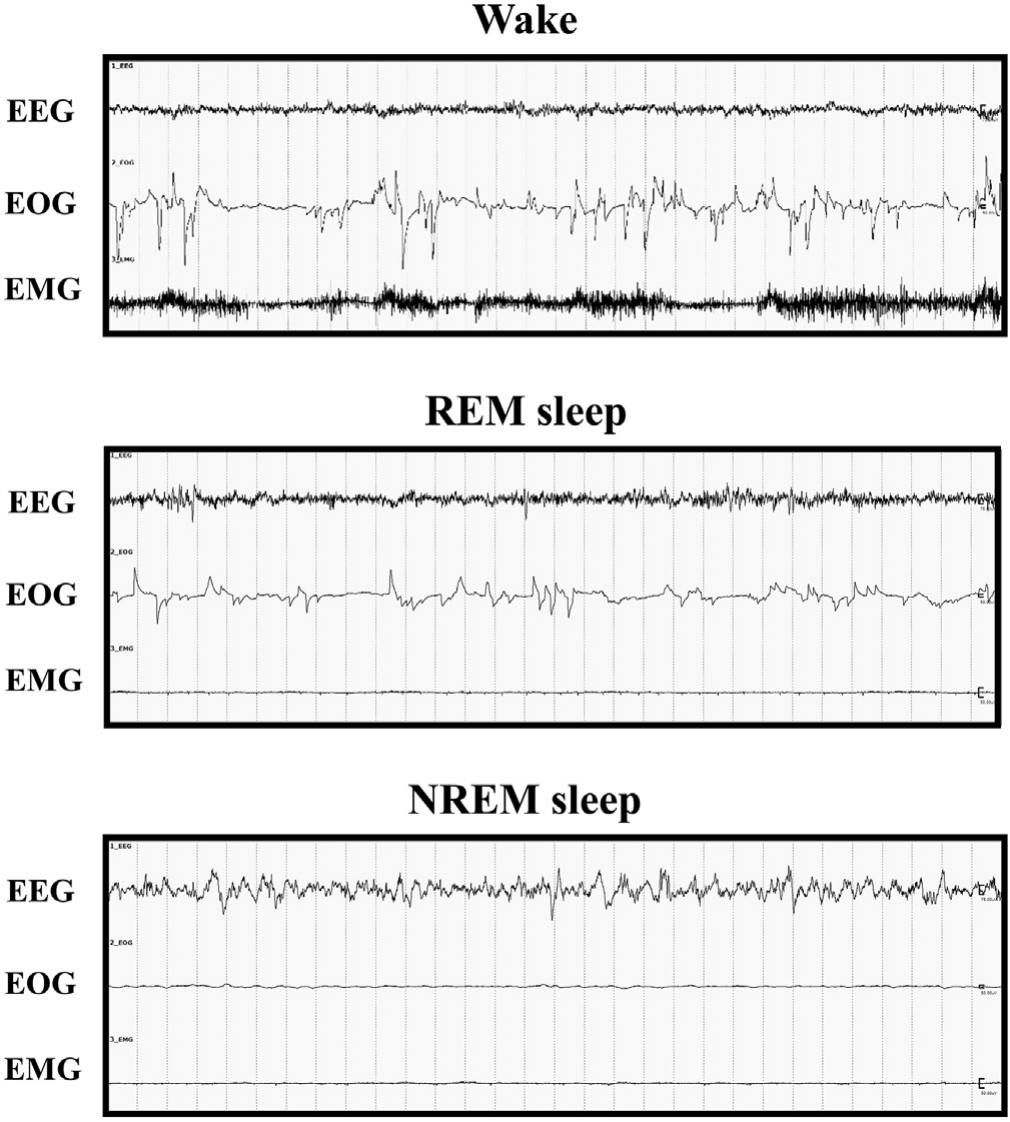
Recordings of wake and sleep stages in cynomolgus monkeys. The charts represent samples recorded over 30-s epochs. Wake, awake stage; REM, rapid eye movement; NREM, non-REM; EEG, electroencephalogram; EOG, electrooculogram; EMG, electromyogram.

The recorded data from the telemetry system were analyzed using SleepSign software (version 3; Kissei Comtec Co., Ltd.). The duration of the wake state was separated into 1-h increments. The vigilance states of the monkeys were identified over 30-s epochs, and three different states were identified: wakefulness, NREM sleep, and REM sleep (Figure 2). Wakefulness was characterized by low-amplitude, high-frequency EEG activity accompanied by high-amplitude EOG and EMG activities. In the sleep state, EEG traces of brain wave activity have revealed that NREM sleep is characterized by very slow but relatively high-amplitude or high-voltage oscillations (with the frequency gradually slowing and the amplitude increasing as sleep deepens), whereas REM sleep has a much faster and lower amplitude trace, similar to normal waking activity. EMG traces of skeletal muscle activity show that although the body is effectively paralyzed during REM sleep, the body does make limited movements during NREM sleep. EOG traces indicate that little or no eye movement occurs during NREM sleep and rapid eye movements occur during REM sleep.

### 2.6 Locomotor activity measurement using the Nano-Tag system

Locomotor activity was measured as cross-count data using the Nano-Tag system. First, a 3-axis composite wave was generated from the X, Y, and Z values measured by a Nano-Tag 3-axis accelerometer. For each recording interval, the number of moments crossing the 3-axis composite wave threshold was recorded as locomotor activity [21]. The threshold was set to 100. Subsequently, we obtained a measure of locomotor activity using the Nano-Tag/Viewer program (Kissei Comtec Co., Ltd.). The activity counts were separated into 1-h increments.

### 2.7 Drugs

The drugs used were modafinil (Modiodal; Alfresa Pharma Co.) and triazolam (205-14221; Fujifilm Wako Pure Chemical Corp., Osaka, Japan). The drug doses used in this study were the same as those recommended in previous reports [22, 23, 24, 25]. Each drug was suspended in 0.5% (w/v) methylcellulose (131-17811; Fujifilm Wako Pure Chemical Corp.). The modafinil or a vehicle was injected with a nasogastric tube 30 min before the dark period (at 18:30). The triazolam or a vehicle was injected with a nasogastric tube 3 h after the lights were turned on (at 10:00). Each animal was given a single dose of one drug/vehicle (only) on each experimental day. Each drug or vehicle was administered at intervals of at least 7 days.

### 2.8 Data analysis

Data are presented as the means ± standard errors. All statistical analyses were performed using GraphPad Prism software (version 6; GraphPad Software Inc., La Jolla, CA, USA). Pearson's product-moment correlation coefficient was used to measure correlations between the awake time per unit time measured via polysomnography and locomotor activity counts per unit time measured via Nano-Tag. Pharmacological data were analyzed using analysis of variance followed by Dunnett's test for multiple comparisons between groups, if appropriate. A *p-*value < 0.05 was considered significant.

## 3 Results

### 3.1 Effects of surgery and suitable implantation sites for the Nano-Tag device

To assess the effects of subcutaneous implantation surgery (n = 2), Nano-Tag measurements were continuously collected over the dark period from the day of surgery (Day 0) to 6 days after surgery (Day 6), and also measured 14 days after surgery. In a previous study, the influence of surgery was examined for 14 consecutive days after implanting Nano-Tags subcutaneously in the backs of cynomolgus monkeys [26]. The results revealed that locomotor activity did not change significantly between days 3 and 14 after surgery (day 0). Our data during the 12-h dark phase revealed that there were similar counts of locomotor activity between day 3 (animal #1: 13,958 counts/12 h, animal #2: 9,434 counts/12 h) and day 6 (animal #1: 13,247 counts/12 h, animal #2: 9,242 counts/12 h) after the tag was implanted in the forehead, although high counts were recorded on the day of surgery as well (day 0; animal #1: 30,666 counts/12 h, animal #2: 16,526 counts/12 h).

To examine the most suitable position for implanting the Nano-Tag (n = 1), Nano-Tags were subcutaneously implanted in the forehead, back, chest, abdomen, and upper right arm of a monkey after surgical implantation of the telemetry system (Figure 1). We collected data on day 16 after the surgery and examined the correlation between changes in awake time as measured by polysomnography using telemetry and locomotor activity counts as measured by the Nano-Tag. Although high correlation coefficients were recorded from 19:00 until 19:00 the next day (24-h duration) for all five sites (forehead: r = 0.898, back: r = 0.883, chest: r = 0.874, abdomen: r = 0.828, and upper right arm: r = 0.885), the correlation coefficient for the forehead had the highest value.

### 3.2 Correlations between awake time per unit time as measured using polysomnography and locomotor activity counts per unit time as measured using the Nano-Tag

**Figure 3:**
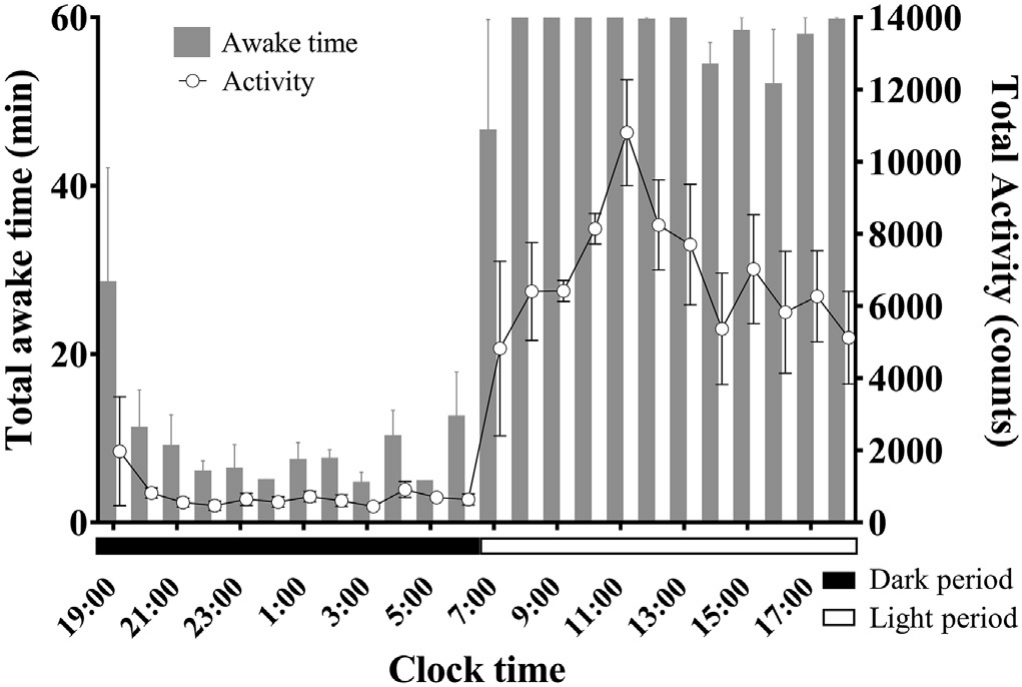
Hourly awake times and locomotor activity counts in cynomolgus monkeys (n = 3) over one day. Error bars denote standard errors.

Based on a preliminary study, we statistically examined the correlations between awake time per unit time as measured using polysomnography and locomotor activity counts per unit time as measured using the Nano-Tag at 9 days after the surgery (n = 3). The awake time per unit time was calculated over 24 h of polysomnographic measurements, from 19:00 until 19:00 the next day. The results were very similar to observations of locomotor activity per unit time as measured using the Nano-Tag (Figure 3). The diurnal preference of cynomolgus monkeys was confirmed. The sleep–wake state pattern confirmed that the monkeys were predominantly awake during the 12-h light period, with naps taking place during the latter half of the light period. Animals quickly fell asleep at the beginning of the dark period. Sleep was predominant during the 12-h dark period and showed a progressive decrease at 1–6 h after the lights were turned off, followed by a progressive increase at 6–12 h after the lights were turned off. Aggressive activity was detected during the 12-h light period and peaked at 5 h (10,811.7 ± 1,467.5 counts/h) after the lights were turned on. Although there was a progressive decrease at 6–12 h after the lights were turned on, a second peak was detected at 9 h (7,025.8 ± 1,509.6 counts/h) after the lights were turned on. Low counts of locomotor activity were observed during the 12-h dark period. The correlation coefficient for the 24-h period was 0.950 (*p* < 0.01). However, although just before the dark period transitioned to the light period, awake time per unit time as measured using polysomnography increased, whereas locomotor activity counts per unit time as measured using the Nano-Tag decreased. Video-based observations showed little to no locomotor activity just before the transition from the dark period to light period.

### 3.3 Comparisons of awake time and activity counts when sleep-disorder treatment drugs were administered

**Figure 4:**
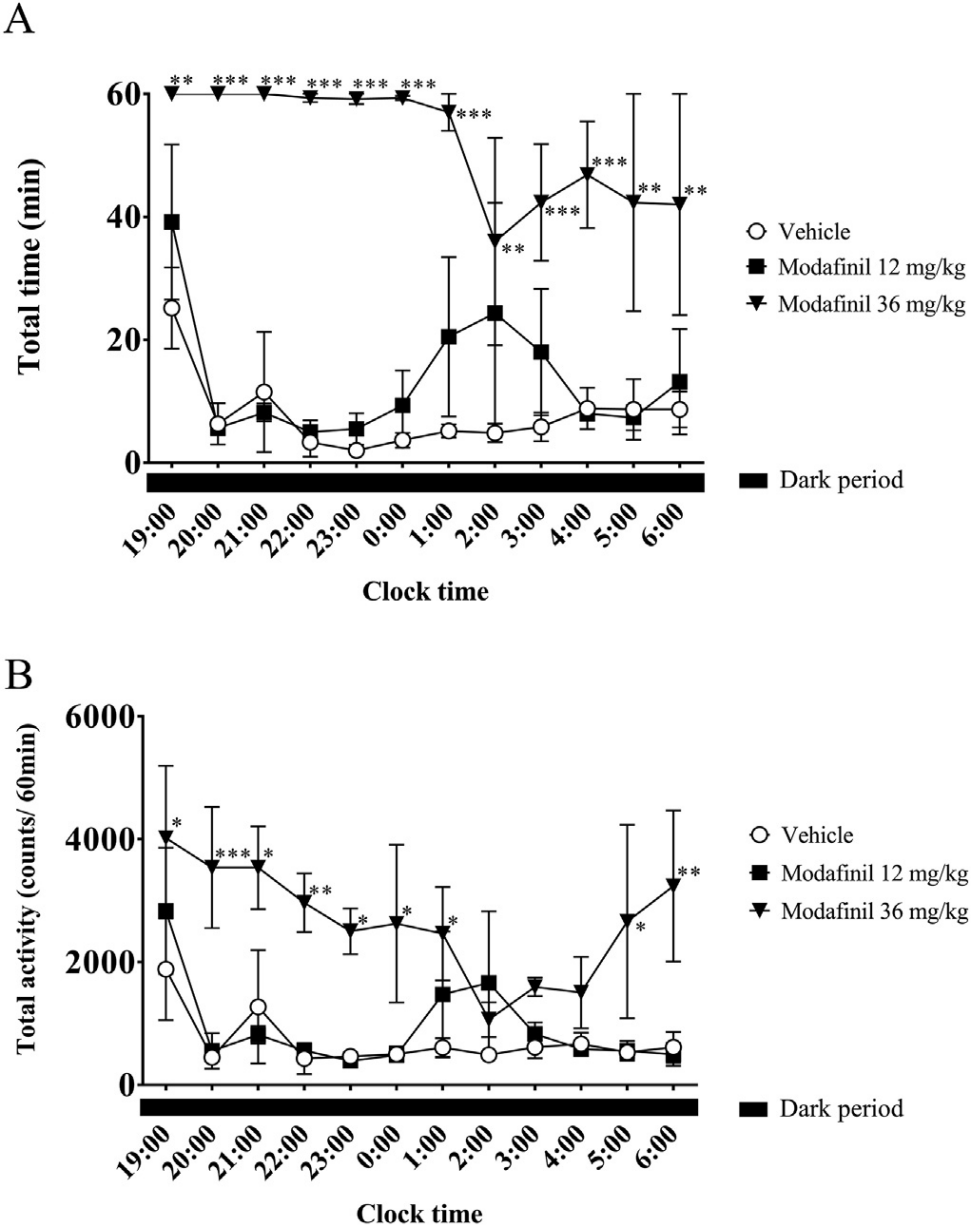
Hourly awake time (A) and locomotor activity counts (B) in modafinil-injected cynomolgus monkeys (n = 3) during the dark period. ∗, *p* < 0.05; ∗∗, *p* < 0.01; ∗∗∗, *p* < 0.001, compared with the control.

We proceeded with our investigation by administering sleep-disorder treatment drugs with proven efficacy to the cynomolgus monkeys (n = 3). We compared the changes in awake time per unit time as measured using polysomnography and locomotor activity counts per unit time as measured using the Nano-Tag after administering the hypersomnia treatment drug modafinil. Modafinil (12 or 36 mg/kg) or a vehicle was injected with a nasogastric tube 30 min before the dark period (at 18:30). In the 12-mg/kg group, wakefulness during the first half of the dark period was relatively similar to that in the vehicle group, indicating that the sleep state was predominant (Figure 4A). Wakefulness showed a tendency to increase at 6–9 h after the lights were turned off, followed by a progressive decrease at 10–12 h after the lights were turned off. In the group treated with 36 mg/kg modafinil, wakefulness increased significantly compared to the vehicle group, and the dominance of wakefulness persisted during the 12-h dark period. A similar pattern was observed for locomotor activity in each group using data from telemetry (Figure 4B). The correlation coefficients during the 12-h dark period were all positive for each group; the coefficients for the vehicle group, 12-mg/kg group, and 36-mg/kg group were 0.937 (*p* < 0.001), 0.953 (*p* < 0.001), and 0.663 (*p* < 0.05), respectively.

**Figure 5:**
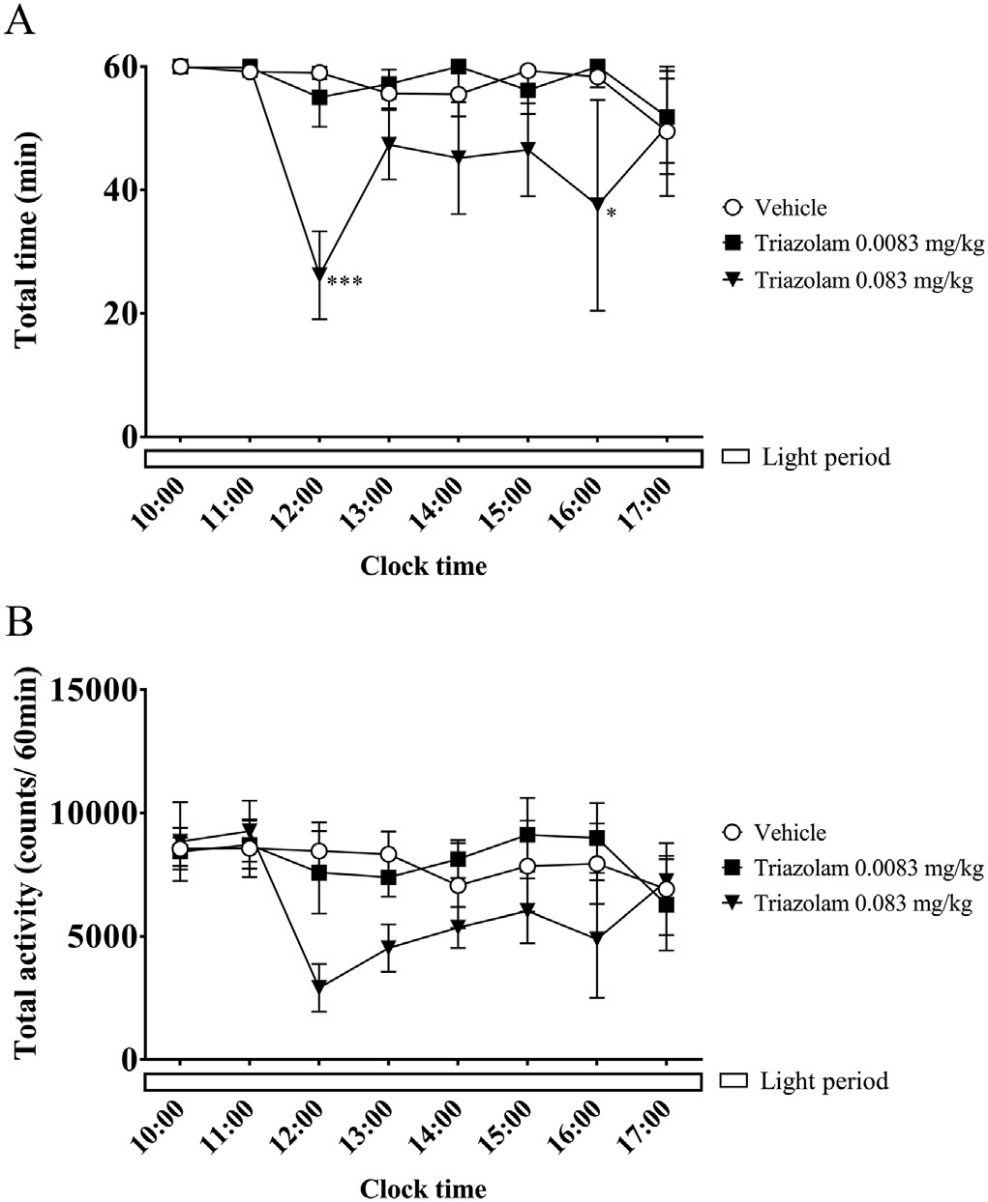
Hourly awake time (A) and locomotor activity counts (B) in triazolam-injected cynomolgus monkeys (n = 3) during the light period. ∗, *p* < 0.05; ∗∗, *p* < 0.01; ∗∗∗, *p* < 0.001, compared with the control.

We also compared the changes in awake time per unit time as measured using polysomnography and locomotor activity counts as measured using the Nano-Tag after administering the insomnia treatment drug triazolam. Triazolam (0.0083 mg/kg or 0.083 mg/kg) or a vehicle was injected with a nasogastric tube at 3 h after the lights were turned on (at 10:00). In the group treated with 0.0083 mg/kg triazolam, the sleep–wake state pattern observed confirmed that wakefulness was predominant during the 12-h light period (Figure 5A). In the group treated with 0.083 mg/kg triazolam, awake time decreased at 3–7 h after administration. Similar patterns were observed for locomotor activity using the telemetry data. The results indicated that low activity tended to be detected at 3–7 h after administration in the group treated with 0.083 mg/kg triazolam (Figure 5B). The correlation coefficients during the 12-h light period were all positive for each group; the coefficients for the vehicle group, 0.0083 mg/kg group, and 0.083 mg/kg group were 0.779 (*p* < 0.05), 0.741 (*p* < 0.05), and 0.931 (*p* < 0.01), respectively.

## 4 Discussion

Sleep disorders typically co-occur with a variety of mental and neurological disorders, such as depression, anxiety disorders, and dementia [27, 28, 29]. Appropriate animal models help with a better understanding of the regulation of the sleep–wake cycle, and the mechanisms underlying sleep disorders. EEG, EMG, and EOG signals and recordings are costly to obtain and complex to analyze. In addition, scoring the sleep and wake stages requires patience and time. It is not a desirable method for a rapid drug efficacy assessment system because the test duration is limited to a few days, and it takes a long time before results can be analyzed fully. By contrast, an implantable locomotor activity measuring device called the Nano-Tag can be subcutaneously implanted during a relatively easy surgery. It takes a short time to obtain results from the analysis using software, and the cost to obtain the data is much lower than that for telemetry. In this study, we examined whether data on locomotor activity as measured using the Nano-Tag would be useful for identifying the sleep–wake stage and screening sleep-disorder drugs. As a preliminary study, we examined when the Nano-Tag enabled measurement of locomotor activity after surgery and determined the most suitable position to implant the Nano-Tag. To investigate postoperative effects, we monitored locomotor activity after surgery. Cynomolgus monkeys typically show low activity during the dark period. However, dark period activity increased transiently following surgery. This likely resulted from pain or discomfort at the surgical site. In the previous study, there was no effect of surgery on locomotor activity at 3 days after Nano-Tags were implanted subcutaneously into the backs of cynomolgus monkeys [26]. Our results also revealed that locomotor activity counts were similar between days 3 and 14 after subcutaneous Nano-Tag implantation in the foreheads of cynomolgus monkeys. The minimally invasive surgery and small skin incision associated with Nano-Tag implantation did not appear to effect locomotor activity measurements at 3 days after surgery. Therefore, we assessed the correlation between awake time per unit time as measured using polysomnography and locomotor activity counts per unit time as measured using the Nano-Tag 9 days after implantation surgery. Next, we implanted locomotor activity devices subcutaneously at five locations (forehead, back, chest, abdomen, and right upper extremity) in a cynomolgus monkey embedded with EEG, EMG, and EOG electrodes and a transmitter, and we identified the most highly-correlated implantation site by comparing awake time calculated from telemetry data and locomotor activity counts at the five implantation sites. Although all five sites produced high correlation coefficients, the coefficient at the forehead was the highest. The forehead of an unrestrained monkey reared in a cage was the easiest site to read with a card reader. Thus, we decided to use the forehead as the implant site for locomotor activity measurements during the sleep experiments.

Subsequently, we implanted Nano-Tags subcutaneously into the foreheads of three cynomolgus monkeys to examine the correlation between changes in awake time as measured via polysomnography using telemetry data and locomotor activity counts as measured using the Nano-Tag along with the correlation between changes in awake time and locomotor activity counts. The awake time per unit time was calculated over 24 h of polysomnographic measurements taken from 19:00 until 19:00 the next day. The results were very similar to observations of locomotor activity per unit time as measured using the Nano-Tag; the correlation coefficient was 0.950 (*p* < 0.01). However, just before the dark period transitioned to the light period, awake time per unit time as measured using polysomnography increased, whereas locomotor activity per unit time as measured using the Nano-Tag decreased. The video observation showed that the monkeys were either stationary or moved slightly in this period. These results suggest that animals were awake during this period, but they did not move much, which may have been the reason for the increase in awake time and decrease in locomotor activity. In spite of this result, we confirmed that changes in awake time per unit time could be determined easily using data on locomotor activity measured via the Nano-Tag. Then, we proceeded with our investigation by administering sleep-disorder treatment drugs with proven efficacy. When we compared the changes in awake time per unit time as measured using polysomnography and locomotor activity per unit time as measured using the Nano-Tag after administering the hypersomnia treatment drug modafinil, the correlation coefficients during the dark period were positive When we compared the changes in the awake time per unit time and locomotor activity after administering the insomnia treatment drug triazolam, the correlation coefficients during the light period were also positive. The analyses of EEG, EMG, and EOG were classified visually from the SleepSign software waveform displays. The results of locomotor activity were calculated by the Nano-Tag/Viewer program. In our study, an automatic algorithm was not used to analyze the EEG, EMG, EOG, or accelometric data. Although the automatic algorithm enables rapid classification, the output often contains errors. To avoid errors associated with automatic algorithms, we assessed all results visually. These results suggest that the measurement of locomotor activity via the Nano-Tag can be used to screen drug efficacy and quickly evaluate sleeping patterns on a long-term basis after surgery with less burden on the animal subjects.

Compared to the telemetry system, the Nano-Tag is relatively inexpensive with a low burden on animals because it is a smaller device and easy to implant. The efficiency of the Nano-Tag allows for reduced sample sizes and is associated with animal welfare. The Nano-Tag is a data logger type measuring device that stores data in the memory inside the main unit. Therefore, unlike telemetry, it is not possible to see the data in real time. The data stored in the internal memory are read via short-range wireless communication using a FeliCa reader within 1 cm of the implant. The Felica reader is applied to the implant for a few minutes until the data reading is complete. Thus, the animal was held in a minimally invasive manner during the data read. The device has a long consecutive measurement time of approximately 60 days [21]. Furthermore, an analysis using rats determined that there was a high correlation between changes in awake time per unit time as measured using EEG and EMG electrodes and locomotor activity as measured using the Nano-Tag (supplemental data). These results suggest that data on locomotor activity obtained via the Nano-Tag can be used to identify sleep–wake states on a long-term basis with less burden on animal subjects. It is impossible for the Nano-Tag to detect sleep depth (non-REM sleep stage), sleep type (non-REM sleep or REM sleep), and sleep quality (change of delta power by EEG frequency analysis) derived from polysomnographic measurement. However, it can detect sleep or wake stages. Nano-Tag measurements would represent a valuable tool for dose response studies and studies aimed at identifying side-effects, such as convulsions, which will inform current clinical studies.

In conclusion, we report that, via measuring locomotor activity, the Nano-Tag is an effective tool for identifying sleep–wake states and screening sleep-disorder drugs with a low cost and less burden on animal subjects.

## Declarations

### Author contribution statement

Keita Sakai: Conceived and designed the experiments; Performed the experiments; Analyzed and interpreted the data; Contributed reagents, materials, analysis tools or data; Wrote the paper.

Akiyoshi Ishikawa, Takehiro Maki: Performed the experiments; Analyzed and interpreted the data; Contributed reagents, materials, analysis tools or data.

Yuri Mizuno: Analyzed and interpreted the data; Contributed reagents, materials, analysis tools or data.

Yasuhiro Oda, Eiki Takahashi: Analyzed and interpreted the data; Wrote the paper.

### Funding statement

This work was supported by the research fund of HAMRI Co., Ltd.

### Competing interest statement

The authors declare no conflict of interest.

### Additional information

No additional information is available for this paper.

## Figures and Tables

**SD_fig1_HELIYON-D19-01649 R1 20191213:**
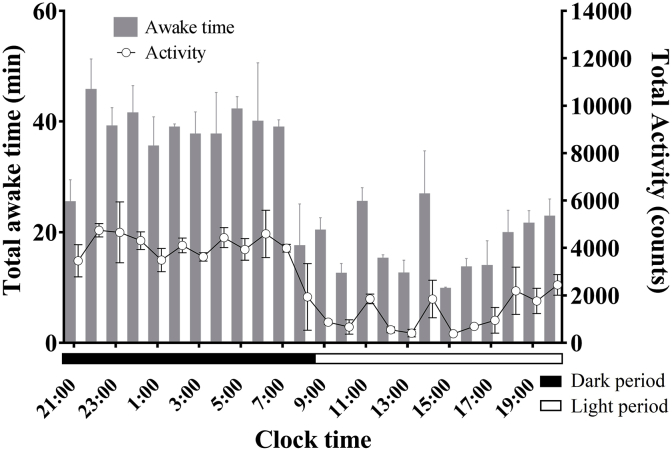


## Appendix A Supplementary data

SD-HELIYON-D19-01649 R1 20191213 SD-HELIYON-D19-01649 R1 20191213
