# Supplementary material for: Simplified drug efficacy screening system for sleep-disorder drugs using non-human primates
Source: Heliyon. 2020 Mar 4;6(3):e03524. doi: 10.1016/j.heliyon.2020.e03524 (PMC7058904; doi:10.1016/j.heliyon.2020.e03524)
Supplement: SD-HELIYON-D19-01649 R1 20191213 [file mmc1.docx]

**Supplemental data**

**Materials and Methods**

***Animals***

Sprague-Dawley rats from Charles River Japan (Kanagawa, Japan) were housed in individual cages (RC2016, W250 × H360 × D360 mm, Shintoyo Seisakusho Ltd., Saitama, Japan) maintained at 24 ± 3°C and 55 ± 25% humidity under a 12-h light/dark cycle (light period: 9:00-21:00 h). Rats were allowed *ad libitum* access to tap water and food pellets (CRF-1, Oriental Yeast Co., Ltd., Tokyo, Japan).

***Electroencephalogram (EEG) and electromyogram (EMG) electrodes and Nano-Tag surgery***

Surgeries to implant the EEG and EMG electrodes into male rats (9 weeks old, 295–408 g body weight) were performed. The rats were anesthetized with a combination of 0.3 mg/kg medetomidine (Domitor, Nippon Zenyaku Kogyo Co., Ltd., Tokyo, Japan), 2 mg/kg midazolam (Sandoz Co., Ltd., Tokyo, Japan), and 2.5 mg/kg butorphanol (Vetorphale, Meiji Seika Pharma Co., Ltd., Tokyo, Japan) and stabilized in a stereotaxic apparatus (SR-6R, NARISHIGE Group, Tokyo, Japan). A midline incision was made to expose the skull. Four small holes were drilled in the skull. A reference electrode (anterior/posterior to bregma, +2.5 mm; lateral to midline, -2.0 mm, Unique Medical Co., Tokyo, Japan) and three EEG electrodes (anterior/posterior to bregma, +2.5 mm; lateral to midline, +2.0 mm; anterior/posterior to bregma, −2.0 mm; lateral to midline, +0.5 mm; anterior/posterior to bregma, −4.0 mm; lateral to midline, +3.0 mm, Unique Medical Co.) were placed through the skull on the cortices for the EEG recordings. Each electrode was fixed to the skull using dental acrylic resin (GC Co., Tokyo, Japan) for protection and insulation. In addition, two stainless-steel hook electrodes were inserted into the cervical portion of the trapezius muscle for the EMG recordings. Nano-Tags (ACOS. Co., Ltd, Nagano, Japan) were implanted in the backs of the rats immediately after implanting the EEG and EMG electrodes. For each rat, the back skin was incised, a Nano-Tag was implanted subcutaneously, and the skin was sutured with suture thread (Alfresa Pharma Co., Osaka, Japan). At the end of the surgery, 20,000 U penicillin G potassium (Meiji Seika Pharma Co., Ltd.) and 0.05 mg/kg buprenorphine hydrochloride (lepetan; Otuka Pharmaceutical Co., Ltd., Tokyo, Japan) were administered subcutaneously. The rats were placed on a 37°C hot pad until they woke up.

***Recording and analysis of* *polysomnographic* and locomotor activity data**

The rats were placed in individual recording cages (RC2016, Shintoyo Seisakusho Ltd.). EEG and EMG signals were amplified (AB-610J, Nihon Kohden Co., Tokyo, Japan), digitized at a sampling rate of 256 Hz, and analyzed using Sleep Sign software (version 3, Kissei Comtec Co., Ltd, Nagano, Japan). The vigilance states in the rats were identified in 8-s epochs, and three different states were identified: wakefulness, non-rapid eye movement (NREM) sleep, and REM sleep. Wakefulness was characterized by low-amplitude, high-frequency EEG activity accompanied by high-amplitude EMG activity. Rats was scored as exhibiting NREM sleep when the EEG data showed high-amplitude and mixed-frequency activity accompanied by low EMG activity. REM sleep was characterized by low-voltage, mixed-frequency activity within the 4–8 Hz range (theta) and accompanied by low or no EMG activity.

The locomotor activity data were collected from Nano-Tag-implanted rats via noninvasive light touches using a FeliCa reader (RC-S360; SONY Corporation, Tokyo, Japan). We obtained measurements of locomotor activity using the Nanotag/Viewer program (Kissei Comtec Co., Ltd.). The activity count was separated into 1-h increments.

***Data analysis***

Data are presented as means ± standard errors of the means. All statistical analyses were performed using GraphPad Prism Software version 6 (CA, USA). Pearson Product-Moment Correlation Coefficient was used to measure correlations between wakefulness and locomotor activity. In all analyses, *p* < 0.05 was taken to indicate statistical significance.

**Results**

We used rats (n = 3) with Nano-Tags implanted subcutaneously in the back to examine the correlation between changes in awake time as measured via polysomnography using EEG and EMG recordings and locomotor activity as measured using the Nano-Tag. The awake time per unit time was calculated from polysomnographic data obtained between 21:00 and 21:00 the next day, and results were very similar to observations of locomotor activity per unit time as measured using the Nano-Tag (Supplemental Fig. 1). Rats are polyphasic sleeping animals that are nocturnal and sleep several times per day. During the 12-h dark period, the sleep–wake state pattern confirmed that wakefulness was predominant over sleep. During the 12-h light period, the sleep–wake state pattern confirmed that sleep was predominant over wakefulness. Locomotor activity data were similar to those obtained via polysomnography. Aggressive activity was detected during the 12-h dark period, and few activity counts were observed during the 12-h light period. The correlation coefficient over these 24 h was 0.955 (*p* <0.001).

**Figure legends**

**Supplemental Fig. 1.** Hourly awake times and locomotor activity counts in rats (n = 3) over one day.
